# Prognosis of Percutaneous Intervention of a Left Main Coronary Artery Stenosis Without the Use of Intravascular Imaging

**DOI:** 10.7759/cureus.2857

**Published:** 2018-06-22

**Authors:** Mazhar Mahmood, Afrasyab Altaf, Momin Salahuddin, Momin Khan, Karamat A Shah, Hammad Shah

**Affiliations:** 1 Department of Cardiology, Rehman Medical Institute, Peshawar, PAK; 2 Cardiology, Tongji Hospital of Tongji University, Shanghai, CHN

**Keywords:** ischemic heart disease, left main stem, intravascular imaging, percutaneous coronary intervention, coronary artery stenosis

## Abstract

Objectives

The aim of this study was to assess the prognosis in patients with left main coronary artery stenosis one year after percutaneous coronary intervention (PCI).

Methods

Our study included 40 patients who underwent PCI for left main coronary artery stenosis without the use of intravascular ultrasound (IVUS). Patients were followed for a year, and the prognostic effect of PCI on a composite end-point of revascularization, new myocardial infarction, cardiac death, and on all-cause mortality was assessed in multivariable Cox analysis.

Results

The multivariable analysis showed a good prognosis in patients receiving PCI with a total event rate of 7.5%. The independent predictors for major adverse cardiac events (MACE) were diabetes (p = 0.02). Other prognostic factors included in the model were gender, age, smoking, body mass index (BMI), hypertension, the complexity of the vessel, and ejection fraction.

Conclusion

PCI for left main coronary artery stenosis without the use of IVUS has a good prognosis after one year of clinical follow-up.

## Introduction

Left main coronary artery disease (LMCAD) is associated with significant morbidity and mortality. The relative risk of perioperative mortality for patients with significant LMCA stenosis compared with patients without LMCAD is 1.3. The five-year mortality in coronary-artery bypass grafting (CABG) patients with three-vessel disease is 10.7%, compared with 15.8% in patients with LMCAD [1-4]. Conventionally, CABG is recommended for most patients with LMCAD [5-6].

However, more recently, randomized trials have shown that percutaneous coronary intervention (PCI) might be an acceptable alternative for such lesions in certain cases [7-10]. This is particularly true in patients with coronary artery disease of low or intermediate anatomical complexity [9]. With recent advances in an improved risk factor profile, careful patient selection, newer drug-eluting stents (DES), and improved intravascular imaging modalities, the use of PCI is expanding.

The aim of this study was an evaluation of clinical outcomes, including unstable angina, myocardial infarction, target vessel revascularization, and death in patients undergoing left main stem stenting without the use of intravascular imaging. Informed consent was taken from all participants in the study which abided by the Declaration of Helsinki.

## Materials and methods

Enrollment, randomization, and follow-up

Patients were assessed for eligibility by interventional cardiologists in collaboration with cardiac surgeons. Inclusion criteria included stenosis of the left main coronary artery of 50% or more, as estimated visually, with a consensus for eligibility for revascularization with either PCI or CABG and patients with a low-to-intermediate anatomical complexity of coronary artery disease (SYNTAX score 32 or less). Exclusion criteria included left main stem disease, along with triple vessel disease, and patients with a high anatomical complexity of coronary artery disease (Synergy Between PCI with Taxus and Cardiac Surgery (SYNTAX) score more than 32).

A history was taken and a detailed examination was done for all patients. Twelve-lead electrocardiography was performed before and after the procedure. Levels of the troponin were measured at the baseline and at 12 and 24 hours after the procedure. Clinical follow-up was performed at one month, six months, and one year. Echocardiography was done at the baseline and then at one year during follow-up. Risk factors were managed according to standard protocols, and guideline-directed medical therapy was recommended for all the patients.

Revascularization strategies and medications

The technique of performing PCI is described in detail elsewhere [11]. Intravascular ultrasonographic guidance was not used. Drug-eluting stents were deployed in all patients. Anticoagulation was achieved with heparin during the procedure and with glycoprotein (GP) IIb/IIIa inhibitors in the initial 12 hours post procedure. Dual antiplatelet therapy was advised for all patients.

Assessment of risk and follow-up for adverse outcomes

A team of cardiologists was involved in the follow-up of patients. Patients were contacted after a year by telephone, as well as scheduled consultations to assess for adverse events. Three patients were lost to follow-up due to change of permanent address and telephone numbers. Outcomes included in major adverse cardiac events (MACE) were cardiac death, death due to other causes, myocardial infarction, unstable angina, and target vessel revascularization (TVR).

Statistics

The distribution of variables was assessed using the Kołmogorov-Smirnov test. Statistical analysis results are expressed as the means ± SD. The t-test and one-way analysis of variance (one-way ANOVA) were performed on normally distributed data. For analysis of nominal data and proportions (hypertension, and smoking), the x2 test was used. Cox proportional hazards analysis were used to identify risk factors for the occurrence of MACE during follow-up. All baseline, demographic, clinical, and angiographic variables were entered into the model. Results are reported as hazard ratios (HRs) and 95% CIs. All statistical tests were two-tailed, and p values were statistically signiﬁcant at < 0.05. All data were analyzed using the Statistical Package for Social Sciences (SPSS) (IBM SPSS Statistics, Armonk, NY), V.20.0 software.

## Results

The mean age of our study sample was 59 ± 13.02 years. For the purpose of PCI, only DES (sirolimus, everolimus, rapamycin, zotarolimus) were used. Out of the 40 patients who underwent PCI for left main stem disease, 27 (67.5%) were men and 13 (32.5%) were women. Ten patients (25%) were smokers, while 30 (75%) were nonsmokers; nine (22.5%) patients were overweight, 14 (35%) had diabetes, and 13 (32.5%) were hypertensive. Four (10%) of the 40 patients in our study had multivessel disease.

No reflow phenomena were found in any of the patients during the procedure. All 40 (100%) patients were given adjunctive treatment with GP IIb/ IIIa antagonists in the catheterization laboratory, as well as for 12 - 24 hours post-procedure. With regard to maintenance therapy after PCI, 100% were receiving aspirin, clopidogrel, and nitrates, 97.5% received beta-blockers, 90% received angiotensin-converting enzyme inhibitors (ACEI), and 45% received diuretics. Baseline characteristics for the study sample are shown in Table 1. 

**Table 1:** Baseline Characteristics of Patients Included in Study ^a^ Expressed as mean in years ^b^ Expresses as mean with standard deviation n: number; BMI: body mass index; ACEI: angiotensin converting enzyme inhibitors

Index	Frequency (n/%)
Age^a^	59
Gender:	
Males	27/67.5
Females	13/32.5
Smoking	10/25
BMI	9/22.5
Diabetes	14/35
Hypertension	13/32.5
Multivessel disease	4/10
Ejection Fraction^b^	45 ± 12
Aspirin	40/100
Clopidogrel	40/100
Beta Blockers	39/97.5
ACEI	36/90
Nitrates	40/100
Diuretics	18/45

**Figure 1:**
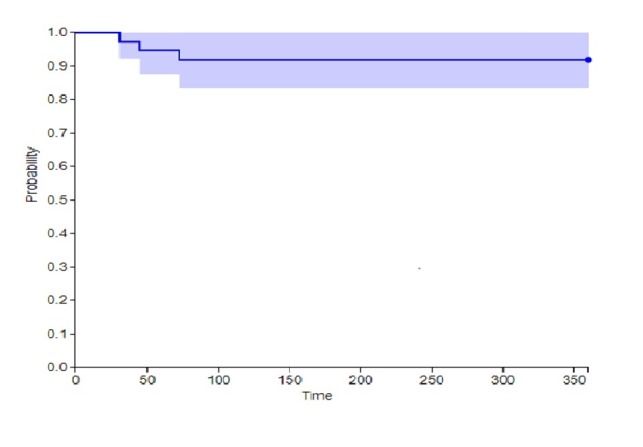
Kaplan Meier analysis of subjects included in study

Cumulative MACE for this study was 7.5% (three patients). The Kaplan Meier analysis is shown in Figure 1.

The one-year mortality was 5% (two patients): myocardial infarction 2.5% (one patient) and unstable angina episodes 2.5% (one patient), and no patient had target vessel revascularization. Among the patients with adverse events, one myocardial infarction patient died.

A multivariate Cox regression analysis with risk factors for coronary artery disease as predictive variables and MACE as the dependent variable were carried out. Analysis showed that diabetes (P = 0.02) was an independent predictor of MACE. Variables excluded by the model were age (P = 0.42), gender (P = 0.75), smoking (P = 0.42), body mass index (BMI) (P = 0.47), hypertension (P = 0.84), ejection fraction (EF) (P = 0.89), and multivessel disease (P = 0.28) as shown in Table 2.

**Table 2:** Cox Regression Table With Hazard Ratio For Various Variables CI: confidence interval; BMI: body mass index

Index	Hazard Ratio (95% CI)	P value
Age	1.18 (0.81 - 1.73)	0.42
Gender	0.94 (0.72 - 1.23)	0.75
Smoking	1.01 (0.99 - 1.03)	0.42
BMI	1.08 (0.84 - 1.38)	0.47
Diabetes	1.51 (1.11 - 2.06)	0.02
Hypertension	0.96 (0.76 - 1.23)	0.89
Multivessel Disease	1.18 (0.91 - 1.52)	0.28
Ejection Fraction	1.03 (0.78 - 1.35)	0.84

## Discussion

Left main stem (LMS) disease has prognostic significance and is found in about 5% of patients admitted for coronary angiographies [12-13]. The Coronary Artery Surgery Study (CASS) showed significantly improved five-year mortality in CABG as compared to medical therapy (16% vs 43%) [14]. CABG is traditionally regarded as the standard treatment for LMS disease, but since the start of PCI era, interventional cardiologists have been rigorously assessing its role in LMS disease.  

The high survival rate for post-CABG patients has been shown in multiple studies [15]. However, the Unprotected Left Main Trunk Intervention Multicenter Assessment (ULTIMA) registry demonstrated promising results for PCI to left main stem with 24% one-year mortality which was even lower in the low-risk group (3.4%) [16]. Biondi-Zoccai et al. [17] also demonstrated a MACE rate of 10.6 and mortality rate of 5.5% for PCI patients. Other registries comparing CABG to PCI also demonstrated similar MACE rates [18-19]. Two important aspects that came to light from these registries were the increased rates of target lesion revascularization (TLR) in the PCI group, while there was a higher incidence of cerebrovascular accidents (CVA) in the CABG patients. The Revascularization for Unprotected Left Main Coronary Artery Stenosis: Comparison of Percutaneous Coronary Angioplasty Versus Surgical Revascularization (MAIN-COMPARE) registry [20] also reported similar results.

The Study of Unprotected Left Main Stenting versus Bypass Surgery (LE MANS) was the first randomized controlled trials (RCT) and enrolled 105 patients with significant LMS disease (defined as > 50% stenosis angiographically) [21]. The primary endpoint was the change in the left ventricular ejection fraction (LVEF) at 12 months, while the secondary endpoint was a major adverse cardiac and cerebrovascular event (MACCE) at 30 days and one year. Surprisingly, there was a statistically significant improvement in LVEF with patients treated with PCI versus CABG (58% versus 54%). PCI was also associated with a lower MACE rate at 30 days (2% versus 13%) with a MACE being equivalent at one year in the two groups. The study did have a number of limitations, including a small sample size, high use of bare metal stents (BMS), and a lower than the contemporary use of left internal mammary artery (LIMA) grafts.

The Synergy Between PCI With Taxus and Cardiac Surgery (SYNTAX) trial was the largest to compare PCI to CABG in LMS disease and demonstrated that if patients were divided into tertiles of syntax score (0-22, 23-32, and above 32), the results were comparable in the lower two tertiles. CABG showed better results as compared to PCI when the syntax score was above 32 [22-23].

Two recent studies showed conflicting results for coronary artery bypass (CABG) vs percutaneous coronary intervention (PCI) in left main coronary artery (LMCA) disease.

The EXCEL (Evaluation of XCIENCE vs Coronary Artery Bypass Surgery for Effectiveness of Left Main Revascularization) trial demonstrated non-inferiority of PCI with everolimus-eluting stents to CABG in low or intermediate SYNTAX score patients [7]. At three years, the MACE rate was 15.4% in the PCI group as compared with 14.7% in the CABG group (95% confidence interval (CI), 0.79 - 1.26) [7].

The NOBLE (Nordic-Baltic-British Left Main Revascularization) trial found that despite a similar mortality, the five-year MACE was higher after PCI as compared to CABG (28.9% for PCI vs 19.1% for CABG) (HR:1.48; 95% CI, 1.11 - 1.96) [24].

Two important factors which could explain the difference between the two trials were the under-utilization of intravascular ultrasound (IVUS) and use of first-generation stents in 11% of PCI patients in the NOBLE trial.

A number of factors can influence the outcome of stenting in left main stem diseases, such as lesion location, stent type, use of intravascular imaging, and complexity of lesion. The distal left main disease poses a particularly challenging vessel anatomy to treat [25]. The Drug-Eluting Stent for Left Main Coronary Artery Disease (DELTA) registry compared ostial/mid-shaft lesions versus distal lesions. The higher rate of TLR was found with distal lesions [26]. In our study, cases with the distal left main disease were relatively fewer, which could explain the good prognosis. Moreover, the Culotte technique appeared to be associated with better outcomes as opposed to the T-stent technique with an in-stent restenosis (ISR) rate of 21% and 56% and a TLR rate of 15% and 56%, respectively [25].

In-stent restenosis and thrombosis are two of the primary concerns in patients coming for PCI. Pandya et al. showed that use of BMS was associated with poorer outcomes than DES [27]. The Intracoronary Stenting and Angiographic Results: Drug-Eluting Stents for Unprotected Coronary Left Main Lesions (ISAR LEFT MAIN and ISAR LEFT MAIN 2) trials were carried out to compare different types of DES [28-29]. These studies proved that outcomes were not influenced by stents (DES) from the same generation.

Appropriate sizing and apposition of stents in LMS disease play an important role in the prevention of ISR and thrombosis. There are no large trials investigating the use of IVUS for PCI but data from certain relatively small studies, such as the MAIN-COMPARE study, point towards improved outcomes with the use of IVUS [30]. In our study, we did not use intravascular imaging due to non-availability of IVUS in our hospital. Patients who agreed to undergo PCI were informed of this limitation. The results in our study were comparable to aforementioned studies, hence showing that absence of intravascular imaging may not be considered a major setback for intervention in left main stem disease.

Limitations

The sample size was relatively small due to stringent inclusion criteria of our study.

Future

In recent times, the outcomes of PCI in LMS disease have improved drastically. This can be attributed to improved stent technology, drug-delivery systems, intravascular imaging, and more potent antiplatelet drugs. More randomized controlled trials (RCTs) are required to establish PCI as a standard treatment option for LMS disease in an era dominated by CABG as the gold standard.

## Conclusions

Percutaneous coronary intervention to left main coronary artery stenosis without the use of intravascular imaging showed good prognosis. It would not only save a huge amount of time for physicians during procedures but also prevent a financial burden on patients if they cannot afford intravascular imaging. Hence, more patients will benefit from left main coronary artery interventions, which are considered high-risk by interventionists. Further studies with large sample sizes and longer follow-up will be required to properly ascertain a full prognosis.
